# Cartilage Degeneration at Symptomatic Persistent Olecranon Physis in Adolescent Baseball Players

**DOI:** 10.1155/2014/545438

**Published:** 2014-12-18

**Authors:** Tetsuya Enishi, Tetsuya Matsuura, Naoto Suzue, Yoshinori Takahashi, Koichi Sairyo

**Affiliations:** Department of Orthopedics, Tokushima University Hospital, 2-50-1 Kuramoto, Tokushima 770-8503, Japan

## Abstract

*Background*. Elbow overuse injuries are common in adolescent baseball players, but symptomatic persistent olecranon physis is rare, and its pathogenesis remains unclear. *Purpose*. To examine the histopathological and imaging findings of advanced persistent olecranon physis. *Methods*. The olecranon physes of 2 baseball pitchers, aged 14 and 15 years, were examined by preoperative magnetic resonance imaging (MRI), and surgical specimens were examined histologically. *Results*. T2-weighted MRI revealed alterations in the intrachondral signal intensity possibly related to collagen degeneration and increased free water content. Histological findings of specimens stained with hematoxylin-eosin showed complete disorganization of the cartilage structure, hypocellularity, chondrocyte cluster formation, and moderately reduced staining. All these findings are hallmarks of osteoarthritis and are suggestive of cartilage degeneration. *Conclusion*. Growth plate degeneration was evident in advanced cases of symptomatic persistent olecranon physis. These findings contribute to understanding the pathogenesis of this disease.

## 1. Introduction

Elbow pain is a common complaint in baseball players and is related to strain from repetitive throwing motion. Elbow pain caused by persistent olecranon physis is relatively rare compared with other causes including medial epicondylitis, osteochondrosis of the humeral capitulum, ulnar nerve neuritis, and other soft tissue injuries [1, 2]. Persistence of the olecranon physis is thought to be caused by valgus extension overload of the ulnohumeral joint, repetitive abutment of the olecranon into the olecranon fossa, traction from the triceps during the deceleration phase of throwing, and impaction of the medial olecranon onto the medial wall of the olecranon fossa [3, 4].

Matsuura et al. presented radiographic criteria for managing symptomatic persistent olecranon physis in adolescent throwing athletes [5], and their criteria have been useful for guiding treatment. However, it is difficult to assess the condition of the physis based on radiographic findings alone. Although magnetic resonance imaging (MRI) can be used to evaluate the condition of the physis precisely, no MRI findings of this lesion have been reported. Furthermore, no consensus exists on the pathology of the physis. Pavlov et al. reported two areas of reactive new bone formation separated by a dense cellular band of collagenous connective tissue without persistent growth plate elements in the lesion [6], while Suzuki et al. showed a widened growth plate with smooth sclerotic borders and round inferior margins [7]. These findings led us to perform MRI and histopathological examination to determine whether or not the lesion is occupied by growth plate remnants or fibrous tissue [2, 3, 6, 7].

Here, we present MRI and histological evidence of cartilage degeneration in 2 cases of persistent olecranon physis. Our findings suggest that the repetitive strain of throwing during sports activity induced the cartilage degeneration at the olecranon physis.

## 2. Method

### 2.1. Case Presentation

Persistent olecranon physis in two male baseball pitchers, aged 14 years and 15 years, was retrospectively evaluated. Both players had experienced elbow pain with restricted elbow extension and tenderness over the olecranon. The physeal lesions were classified as stage II, characterized by sclerotic change, according to radiographic criteria [5]. Operative treatment went ahead when no improvements were seen with conservative therapy including avoiding heavy use of the elbow, such as in throwing, batting, arm wrestling, and carrying heavy loads for at least 3 months. The physis was partially removed for histological examination, and a remnant of the isolated physis was inverted and replanted in the original position. Internal fixation of the persistent physis was achieved with Kirschner wires and a figure-of-eight tension band. Six months after the operation, both patients had regained full normal range of motion and were able to return to pitching activities without pain. A radiograph of the olecranon is shown in Supplementary Figure 1 (see Supplementary Material available online at http://dx.doi.org/10.1155/2014/545438).

### 2.2. Magnetic Resonance Imaging

MRI was performed using a Signa Excite HD 1.5T scanner (GE Yokogawa Medical Systems, Tokyo, Japan).

### 2.3. Histological Examination

Surgical specimens were immersed in 4% paraformaldehyde, decalcified with EDTA, and embedded in paraffin. Sections of 4 *μ*m thickness were cut, stained with hematoxylin-eosin, and subjected to immunohistochemistry. After deparaffinization, rehydration, and several washings in phosphate-buffered saline (PBS), the sections were immersed in methanol containing 0.3% H_2_O_2_ for 30 min. Enzyme digestion with 1% hyaluronidase (Sigma, St. Louis, MO) was performed at 37°C for 1 h. The sections were then incubated with antibodies against proliferating cell nuclear antigen (PCNA; dilution 1 : 200, clone PC10, Dakopatts, Copenhagen, Denmark) overnight at 4°C. After several additional washings in PBS, the specimens were incubated with either biotinylated anti-mouse or anti-goat immunoglobulin G (Vector Lab, Burlingame, CA) for 1 h. Antibody binding was visualized using a Vectastain avidin-biotin-peroxidase complex kit (Vector Laboratories, Burlingame, CA) combined with diaminobenzidine tetrahydrochloride according to the manufacturer's instructions. The sections were counterstained with hematoxylin. Cells with brown stained nuclei were regarded as positive for PCNA.

## 3. Results

### 3.1. Magnetic Resonance Imaging

**Figure 1:**
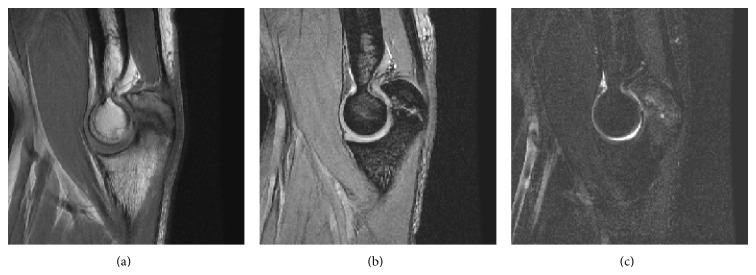
Sagittal T1-weighted (a) and T2-weighted (b) magnetic resonance (MR) images of the right elbow showing abnormal intensity in the olecranon physis. Sagittal T2-weighted MR image with fat saturation (c) of the right elbow showing high intensity in the intramedullary region.

MRI revealed abnormal intensity in the persistent olecranon physis in both cases, which was possibly related to premorphologic intrasubstance collagen degeneration and increased free water content (Figures 1(a)–1(c)). T2-weighted MRI showed intramedullary regions of increased signal intensity reflective of subchondral edema (Figures 1(b) and 1(c)). Upon the comparison with the case in the earlier stage, Supplementary Figure 2 demonstrated the equal intensity signal at the lesion.

### 3.2. Histological Examination

**Figure 2:**
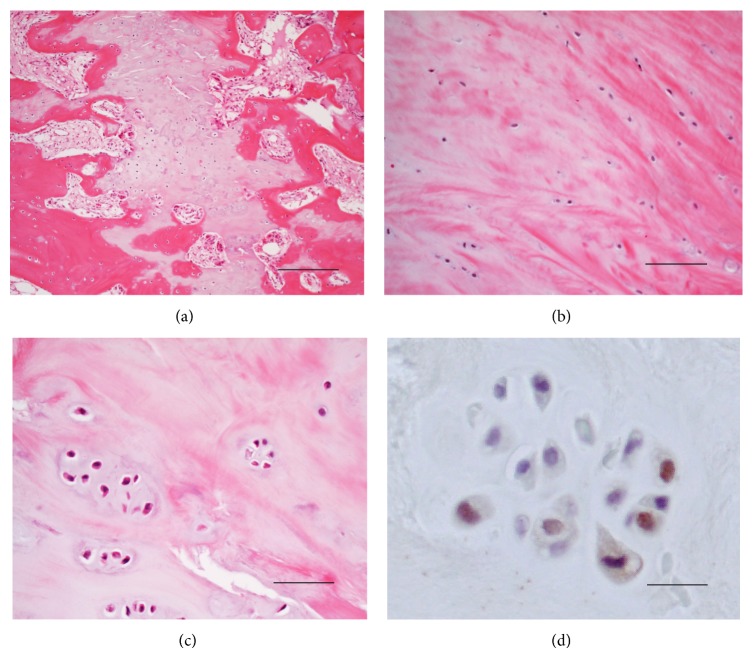
(a) Growth plate remnant with a sclerotic margin. Moderate reduction in hematoxylin-eosin staining and disorientation of chondron columns in the olecranon physis. (b) Magnified view showing a decreased number of chondrocytes in the lesion. (c) Chondrocyte cluster formation in the lesion. (d) Proliferating cell nuclear antigen-positive cells in a chondrocyte cluster. Bars: 200 *μ*m (a), 100 *μ*m (b), 50 *μ*m (c), and 20 *μ*m (d).

Histological examination revealed a sclerotic margin between the epiphysis and metaphysis (Figure 2(a)). Moderately reduced hematoxylin-eosin staining and disorientation of chondron columns were observed at the olecranon physis (Figure 2(a)), and a magnified view showed hypocellularity (Figure 2(b)) with occasional chondrocyte cluster formations (Figure 2(c)). PCNA-positive cells were observed in the chondrocyte clusters (Figure 2(d)) and may have contributed to cluster formation. These findings are consistent with osteoarthritis and strongly suggest cartilage degeneration.

## 4. Discussion

In this report, we have provided evidence of cartilage degeneration in advanced cases of persistent symptomatic olecranon physis in adolescent baseball players. Quantitative evaluation of articular cartilage on MRI is common, especially for evaluating osteoarthritis of the knee [8]. Therefore, we decided to use MRI to examine elbow physis in the present cases. T2-weighted MRI revealed nonuniform signal intensity, which is potentially related to collagen degeneration and increased free water content.

We also presented evidence of cartilage degeneration in both of these cases of symptomatic persistent olecranon physis. Histological findings showed complete disorganization of the cartilage structure, hypocellularity, chondrocyte clusters, and moderate reduction of hematoxylin-eosin staining in the olecranon physes, all of which are well-known hallmarks of osteoarthritis and are strongly suggestive of cartilage degeneration.

Previous research has focused on histological examination of growth plate remnants or fibrous tissue in lesions [2, 3, 6, 7] and revealed the presence of widened physes with sclerotic margins or fibrous tissue without physeal remnants. The differences in histological findings may reflect different disease stages. In light of the present findings, we believe that the pathogenesis of persistent symptomatic olecranon physis could be cartilage degeneration at the growth plate.

Repetitive stress such as throwing is thought to cause symptomatic persistent olecranon physis [3]. The growth plate is biomechanically weak compared with bone and represents the path of least resistance; thus, excessive force may traverse the plate. Isolated extension forces at the triceps insertion, as seen in gymnasts and divers, or combined forces such as valgus extension overload of the elbow of overhead throwing athletes can induce this disease.

The main limitation of this study is that the pathology of the symptomatic physes in the early stage of the disease was not examined. Early-stage lesions were treated conservatively and no specimens were obtained at that time. Therefore, we were unable to determine the natural course of the disease. Another possible limitation is a small number of cases. Further studies are needed to assess the relationship between the cartilage degeneration and persistent olecranon physis and to reveal the natural course of the disease.

## 5. Conclusion

Growth plate degeneration was evident in advanced cases of symptomatic persistent olecranon physis. These findings contribute to understanding the pathogenesis of this disease. This is the first study to provide clear evidence of cartilage degeneration in persistent olecranon physis in adolescent baseball players.

## Supplementary Material

Supplementary Figure 1. a. Lateral radiograph of the right elbow showing persistent olecranon physis. b. Lateral radiograph of the left elbow showing an almost fused olecranon physis. c. Lateral radiograph of the right elbow immediately after the operation. The lesion was fixed with a tension-band wire with 2 pins and a figure-of-eight wire. d. Lateral radiograph of the right elbow 3 months postoperatively showing a fused olecranon physis.
Supplementary Figure 2. a. Sagittal T1-weighted (a) and T2-weighted (b) magnetic resonance (MR) images of the right elbow showing equally intensity in the olecranon physis. Sagittal T2-weighted MR image with fat saturation (c) of the right elbow showing high intensity in the intramedullary region.
